# In-Situ Isothermal Crystallization of Poly(l-lactide)

**DOI:** 10.3390/polym13193377

**Published:** 2021-09-30

**Authors:** Zirui Huang, Meiling Zhong, Haibo Yang, Enqin Xu, Dehui Ji, Paul Joseph, Ri-Chao Zhang

**Affiliations:** 1 School of Materials Science and Engineering, East China Jiaotong University, Nanchang 330013, China; huang_zirui@yeah.net (Z.H.); Xeq0419@163.com (E.X.); jidehui_1990@163.com (D.J.); 2 Anji Municipal Ecology and Environment Bureau, Huzhou 313300, China; yhbhappy2005@163.com; 3 Institute for Sustainable Industries and Liveable Cities, Victoria University, P.O. Box 14428, Melbourne, VIC 8001, Australia; Paul.Joseph@vu.edu.au

**Keywords:** poly(l-lactide), spherulite, nucleation rate, Avrami exponent, morphology

## Abstract

The isothermal crystallization of poly(l-lactide) (PLLA) has been investigated by in-situ wide angle X-ray diffraction (WAXD) and polarized optical microscopes (POM) equipped with a hot-stage accessory. Results showed that the spherulites of PLLA were formed at high temperature, whereas irregular morphology was observed under a low temperature. This can be attributed to the varying rates of crystallization of PLLA at different temperatures. At low temperatures, the nucleation rate is fast and hence the chains diffuse very slow, resulting in the formation of imperfect crystals. On the other hand, at high temperatures, the nucleation rate is slow and the chains diffuse fast, leading to the formation of perfect crystals. The change in the value of the Avrami exponent with temperature further verifies the varying trend in the morphological feature of the crystals.

## 1. Introduction

Poly(l-lactide) (PLLA) is widely used in almost every aspect of modern technologies, including food packaging [1,2], agriculture, biomedicine, etc., [3] owing to its biodegradability and compostability resulting from the readily hydrolyzable ester bonds. Typically, PLLA is a semi-crystalline polymer, and consists of both amorphous regions and crystalline domains. Generally, upon degradation, the chains in the amorphous region start to degrade initially, followed by those in crystalline part [4,5,6]. Therefore, the degradation behavior of PLLA is closely dependent on the morphology and structure of the polymer matrix, which is in turn determined by the crystallization process leading to the formation of the material [7,8]. To gain better control of the crystalline structure and morphology closely related to the nucleation and growth process of PLLA. Therefore, it is highly desirable to understand the crystallization history of the material under isothermal conditions, as this influences the nucleation and growth of the morphological features of ordered regions of the base substance.

Isothermal crystallization behavior of PLLA has been broadly investigated and reported over the past 20 years [9,10,11,12,13,14,15,16,17,18,19,20,21,22]. Typically, PLLA can form different crystal structures through different processing routes. For example, an ordered *α*-phase is produced at high crystallization temperatures [14], while a conformationally disordered *α*’-phase is developed at low temperatures under Quiescent melt, or cold crystallization conditions [23]. Although these two structures have different melting temperatures, they have essentially the same crystal structure with a very minor difference in chain conformation and packing density. Other crystal structures such as *β*-crystals and *γ*-crystals can also be developed, both under the mechanical stretching [12] or solution-spinning [24], and by epitaxial crystallization in hexamethylbenzene [25]. Additionally, the crystallization kinetics of PLLA have also been explored. It is recognized that the growth rate of PLLA crystals decreases as its molecular weight increases [26,27]. Moreover, the regime transition was observed when regime transition theory was used to analyze the isothermal crystallization kinetics. Recently, the same equilibrium melting temperature of both *α*-phase and *α*’-phase has been confirmed through nonlinear extrapolative methods [7], further verifying their identity in crystal structure. Even though some degree of insight into nucleation and growth of PLLA is already established, further studies are definitely warranted in this research area.

In this article, we systematically studied the morphology, structure and isothermal crystallization kinetics of PLLA through in-situ wide angle X-ray diffraction (WAXD) and polarized optical microscope (POM) with a CSS450 hot stage. The nucleation and growth processes were analyzed according to the Avrami theory. Furthermore, the nucleation and growth rates of PLLA can be fine-tuned by adjusting the isothermal crystallization temperature.

## 2. Experimental

### 2.1. Materials and Sample Preparation

Poly(l-lactide) with an average molecular weight (*M_w_*) of 150,000 g/mol supplied by Nature Works LLC, NE, USA, was used as received. Methylene chloride was obtained from Huamei Co., Nanchang, China. The solution casting method was used to fabricate the PLLA films. The pellets of PLLA were first dissolved into methylene chloride to prepare 10 wt.% solution. The solution was then casted onto a glass plate, and then kept at room temperature for 24 h until the solvent was completely evaporated. The PLLA films thus obtained were further dried in vacuum at ca. 50 °C overnight for crystalline morphological observation.

**Figure 1:**
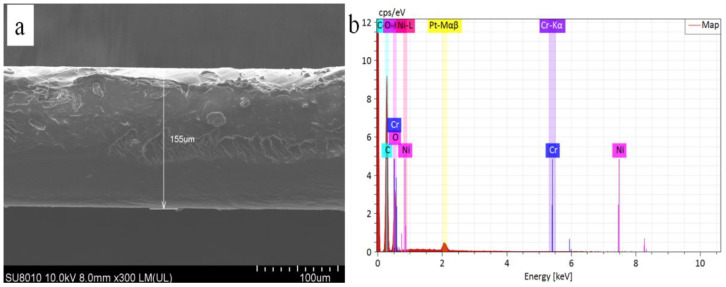
SEM image (**a**) and EDAX (**b**) of PLLA film.

The thickness and elements analysis of films measured by scanning electronic microscope (SEM, HitachiHigh-Technologies Corporation, Tokyo, Japan) are shown in Figure 1. The film shows about 155 um thickness (Figure 1a), and contains a small amounts of the metals such as Cr, Ni and Pt et al. (Figure 1b). The atomic contents of elements are described in Table 1.

### 2.2. DSC and TGA Analysis

The cooling and heating run of PLLA was carried out in a nitrogen atmosphere by using a TA Q100 DSC instrument. The curves were obtained when the samples (ca. 7 mg) were heated to 200 °C at 10 °C/min, held at that temperature for 3 min to ensure complete melting of the polymer, cooled at a rate of 10 °C/min to the 40 °C, and then heated at a rate of 10 °C/min to the melting temperature for further thermal analysis. The TGA analysis of PLLA was performed under a nitrogen atmosphere through a NETZSCH STA 449F5 instrument with a heating rate of 10 °C/min.

### 2.3. In-Situ Isothermal XRD Measurements

The in-situ isothermal crystallization of PLLA was carried out in a nitrogen atmosphere by using a D8 ADVANCE X-ray diffractometer (Bruker Inc., Germany) with Cu Kα radiation. The isothermal crystallization was performed as follows: the samples were heated to 200 °C, held at that temperature for 3 min to ensure complete melting of the polymer, and then cooled at a rate of 30 °C/min to the 100, 110 and 120 °C, respectively, for inducing isothermal crystallization. During the isothermal crystallization, the samples were scattered by XRD at intervals of 1 or 2 min. The data acquisition time was 30 s for each scattering pattern. The X-ray source was set at a voltage of 40 kV. The scattered intensities were registered in the range of scattering angles 2*θ* from 10° to 40°.

The intensity was plotted as a function of the scattering angle, 2*θ*, then through deconvoluting the peaks of linear WAXD profiles, the fraction of material crystallized after a certain period of time was calculated by

(1) $$X_{t}=\frac{\sum A_{cryst}}{\sum A_{cryst}+\sum A_{amorph}}$$

where $A_{cryst}$ and $A_{amorph}$ are the fitted areas of crystal and amorphous, respectively. By plotting *log* [−*ln* (1 − *X*(*t*))] against *log t*, the crystallization half-time, *t*_1/2_, defined as the time taken for half of the crystallinity to develop, was obtained [28].

### 2.4. In Situ Observation of PLLA Crystalline Morphology

The crystalline morphologies of PLLA isothermally crystallized from melt were observed in situ by a polarized optical microscope (POM) equipped with a CSS450 hot-stage accessory. The films of the samples were heated from 20 °C to 200 °C at 20 °C/min, and kept at that temperature for 3 min to allow the completion of the melt, then cooled down to the crystallization temperature at 80 °C/min for inducing isothermal crystallization.

The diameter of the spherulites was recorded as a function of the crystallization time using a calibrated video caliper. The increase of spherulite radius is linearly dependent on the crystallization time until impingement. Under isothermal conditions, the spherulitic growth rate is calculated from the slope of the spherulite diameter vs. time plot.

## 3. Results and Discussion

### 3.1. DSC and DTG Analysis

**Figure 2:**
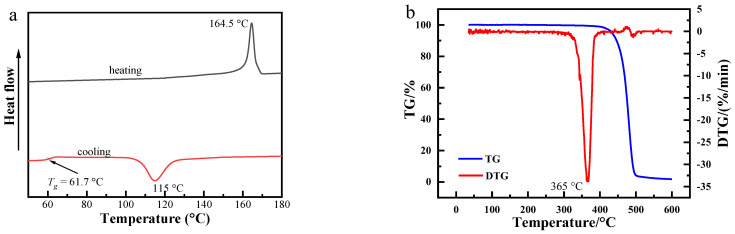
(**a**): DSC cooling and heating curves of PLLA at 10 °C/min; (**b**): the TGA and DTG curves of PLLA.

Figure 2a represents the typical cooling and heating DSC curves of PLLA with a cooling/heating rate of 10 °C/min, respectively. The Tg is about 61.7 °C, the crystallization temperature is about 115 °C and its melting temperature is about 164.5 °C. The TGA and DTG of PLLA samples are shown in Figure 2b. It can be observed that the PLLA starts to degrade at 365 °C, which shows that the PLLA is stable at 200 °C.

### 3.2. Crystalline Morphologies of PLLA

**Figure 3:**
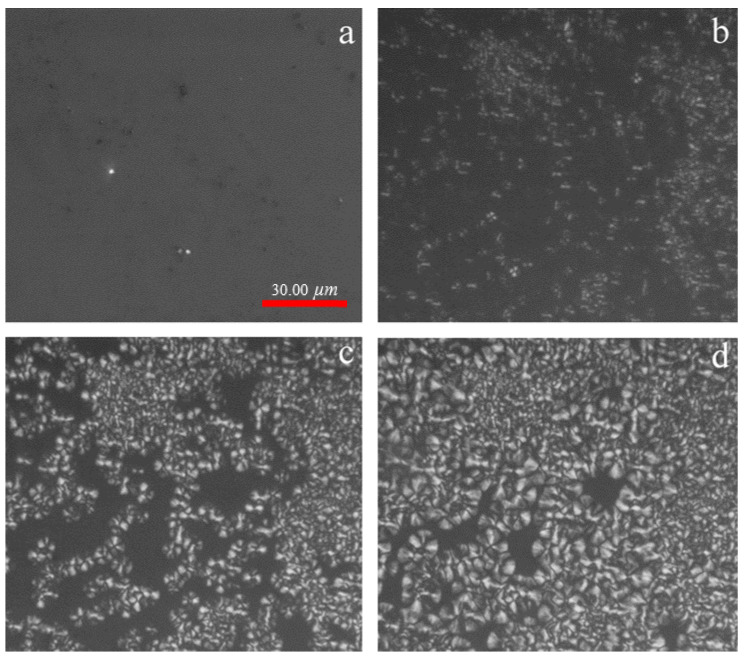
POM of PLLA crystallized isothermally at 100 °C (**a**) 0 min; (**b**) 3 min; (**c**) 6 min; (**d**) 9 min.

With a view to getting a fundamental understanding regarding the type of nucleation and morphology of PLLA, the in situ growing process of spherulite was followed by using POM with a hot stage provision. In Figure 3, the optical microscope photographs of PLLA isothermally crystallized at 100 °C are presented. During the initial stages, PLLA starts to nucleate, and the resulting structures are too small to be observed. As the time elapses, the nuclei grow to form the small spherulites that can be observed under POM (Figure 3b). The small spherulites first formed will further grow in a three-dimensional way, as the melted phase provides enough space for their proliferation. However, as time progresses, the spherulites will start to impinge on each other, thus leaving only a relatively small space for the newly formed nuclei to grow. Therefore, they can only grow in only one- and/or two-dimensional way in the available space. Although the Maltese cross of some spherulites can be observed, most crystals exhibit irregular shapes at the end of crystallization. According to the regime transition theory, at low crystallization temperature the nucleation rate is very fast, while the chains diffuse slowly, resulting in the formation of imperfect crystals.

**Figure 4:**
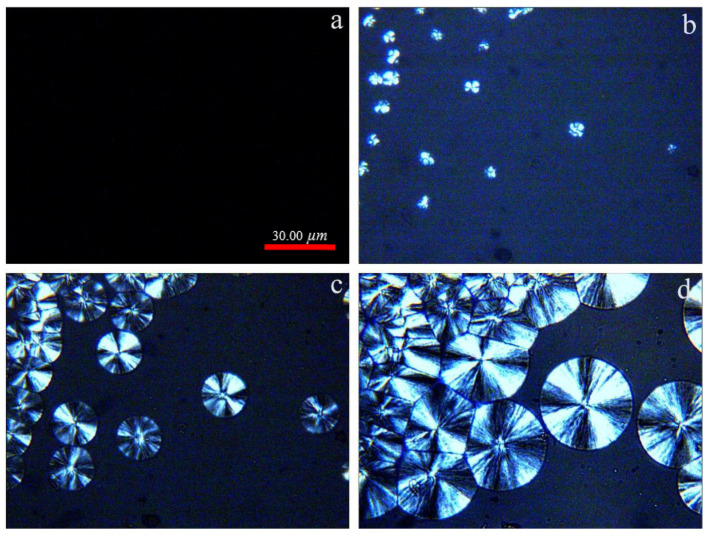
POM of PLLA crystallized isothermally at 120 °C. (**a**) 0min; (**b**) 6 min; (**c**) 16 min; (**d**) 34 min.

Figure 4 represents the POM of PLLA crystallized isothermally at 120 °C for different periods. Typically, the crystalline morphologies of PLLA are spherulites; the Maltese cross can be observed clearly. As the time increases, the diameter of spherulites increases. Compared to the sample crystallized at 100 °C, the number of nuclei is greatly reduced. This result is due to the fact that the chains diffuse very fast, while the nucleation rate is very slow when crystallization takes place at a higher temperature [29,30,31].

### 3.3. Crystal Structure of PLLA

**Figure 5:**
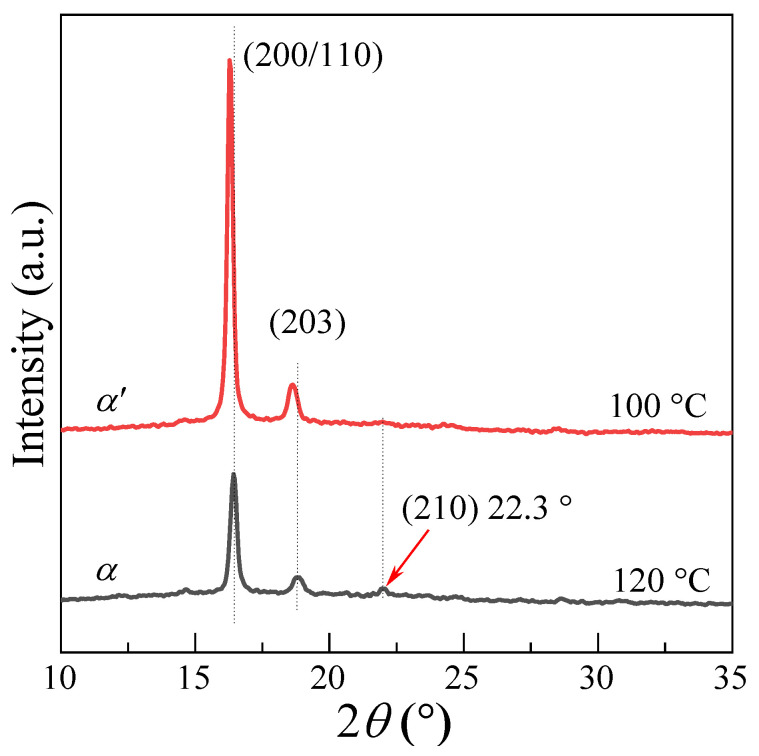
XRD diffraction patterns of PLLA crystallized at 100 °C and 120 °C.

The X-ray diffraction patterns of PLLA samples crystallized at 100 °C and 120 °C are shown in Figure 5. All the diffraction patterns are normalized using the strongest (200)/(110) reflection intensity as the common base. The observed reflections were indexed according to the *α*-crystal phase of the material. Two strong reflection peaks (200)/(110) and (203) can be noticed in both samples. Several weak reflection peaks corresponding to the *α*-phase, such as (010) at 14.8° and (210) at 22.3°, disappear when the crystallization temperature drops from 120 °C to 100 °C. This suggests that crystal structure of PLLA changes from *α*-phase to *α*′-phase. Moreover, the reflection peaks of (200) and (203) shift to a lower scattering angle, thus indicating increased lattice spacing.

### 3.4. Isothermal Crystallization Kinetics of PLLA

The isothermal crystallization kinetics of PLLA was analyzed according to the Avrami equation [32,33,34]

(2) $$1-X\left(t\right)=e^{-Kt^{n}}$$

where *X*(*t*) is the volume fraction of crystals at time, *t*; *K* is the rate constant that includes the temperature dependent terms, and also contains information regarding diffusion and nucleation rates; *n* is the Avrami exponent which depends on the types of processes that occur during nucleation and growth.

However, Lorenzo et al. [35] suggested that direct use of Equation (2) would have encountered many practical problems due to that there exists an initial crystallization time or the induction time, *t*_0_, for the beginning of the crystallization, which is different from the absolute time, *t*. Therefore, they believed that time count must start from time equal to *t*_0_, and a minor modification has to be introduced into the Avrami classical equation, by taking into consideration the experimental induction time *t*_0_. Hence, Equation (2) can be then rewritten as:

(3) $$X\left(t-t_{0}\right)=1-e^{-K{\left(t-t_{0}\right)}^{n}}$$

By taking the logarithm of Equation (3), twice, one can then obtain the isothermal crystallization parameters:

(4) $$ln\left[-ln\left(1-X\left(t-t_{0}\right)\right)\right]=lnK+nln\left(t-t_{0}\right)$$

Subsequently, the double logarithm of amorphous fraction can be plotted against the logarithm of time. Furthermore, the term *X* (*t − t*_0_) can be calculated according to Equation (2) by determining the area under the crystallization isotherm, at each instant, and taking the ratio of the areas at each time interval to the total area. The rate constant, *K*, can be obtained from the intercepts, and *n* from the slope of the line.

Figure 4a–c represents the typical WAXD patterns with crystallization time for PLLA isothermally crystallized at 100, 110 and 120 °C, respectively. It can be observed from Figure 4a–c that only the amorphous peak occurs at the beginning of crystallization time for all samples, and with increasing time, the crystallization peaks can be observed, and the intensity of crystal peaks increases for all the PLLA samples.

**Figure 6:**
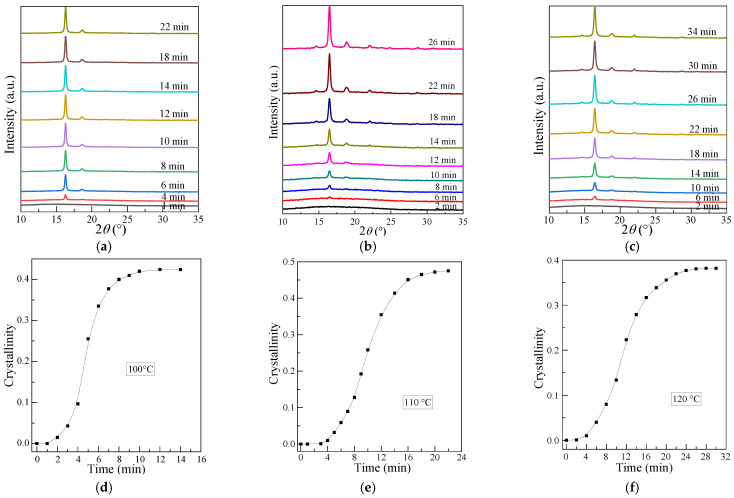
WAXD diffraction patterns of PLLA isothermally crystallized for various time at (**a**) 100 °C; (**b**) 110 °C; (**c**) 120 °C; and (**d**–**f**) are the crystallinity of PLLA calculated from (**a**–**c**), respectively, with crystallization time.

**Figure 7:**
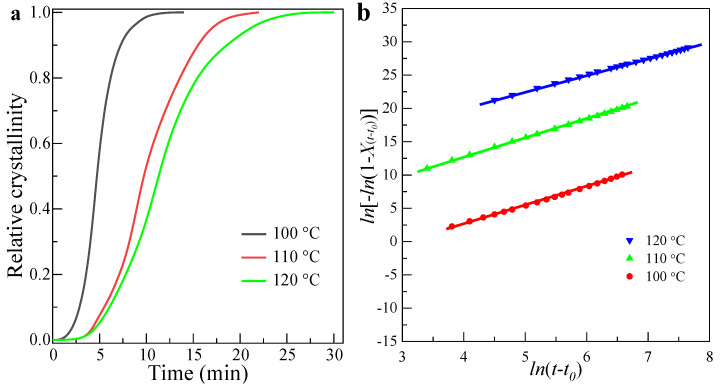
(**a**) Relative crystallinity vs. time (*t* − *t*_0_) of PLLA. (**b**) Double logarithmic plot of amorphous content versus *ln* (*t − t*_0_) (sample crystallized isothermally at 100 °C, 110 °C and 120 °C, respectively).

Based on Equation (1), the calculated crystallinity of PLLA with crystallization time is shown in Figure 6d–f. As can be seen, the crystallinity of PLLA increases with crystallization time and keeps constant at the end of the crystallization. For example, the crystallinity (Figure 6d) of PLLA crystallized at 100 °C for 12 min (Figure 6a) is about 0.42 (± 5%) and invariant as the time increases, indicating that the crystallization process is almost finished. By normalizing the crystallinity with crystallization time, the relative crystallinity, *X*(*t − t*_0_), can be obtained as a function of time, as shown in Figure 7a. It can be also observed that the time required for complete crystallization of PLLA has the identical trend in variation with the crystallization temperature. Figure 7b shows a plot of *ln* [−*ln* (1 − *X*(*t − t*_0_))] versus *ln*(*t − t*_0_) for PLLA isothermally crystallized at different temperatures. The graph was obtained by using the Equation (4). As can be seen from Figure 5, the experimental data of PLLA appear to fit the Avrami equation perfectly, since the Avrami plot is expected to be throughout the entire time period for isothermal crystallization. From Figure 7b, the crystallization parameters from the Avrami equation, such as Avrami exponent *n*, crystallization half time *t*_1/2_, and crystallization rate constant *K* for PLLA are obtained (see in Table 2). It is to be noted here that the value of Avrami exponent *n* decreases from 2.8 to 2.5, as the isothermal crystallization temperature increased from 100 °C to 120 °C. On the basis of this result, it can be clearly inferred that PLLA exhibited homogeneous nucleation, and the crystal growth in a three-dimensional mode when crystallization was carried out at 100 °C. As the crystallization temperature increases, the motion of chains is bound to increase, thereby resulting in a faster growth for the spherulites. After the spherulites grow to a point where they start to impinge on each other, the newly formed crystal nuclei can only grow in a one- and/or two-dimensional way because of space limitation; this results in a decreased value of the Avrami exponent.

It can also be found that the bulk crystallization rate of PLLA, characterized by crystallization half time, *t*_1/2_, decreased from 1133 s to 407 s as the crystallization temperature was decreased from 120 °C to 100 °C, thus indicating a faster crystallization rate for PLLA when crystallized at a lower temperature. The slow crystallization rate at higher temperature shows that the crystallization rate is essentially dependent on the nucleation and growth process. At higher temperatures, the growth rate is faster while the nucleation rate, as determined by the nucleation-free energy, is very slow, resulting in a slower rate of crystallization.

### 3.5. Growth Rate Parameters of Isothermally Crystallized PLLA

The spherulite growth rates (*G*) for PLLA obtained at the temperatures between 100 °C and 150 °C are shown in Figure 6, which have been used to examined in terms of secondary nucleation theory [36]. The general expression for the growth rate of a linear polymer crystal with folded chains is given by

(5) $$G=G^{0}exp\left(\frac{-U^{*}}{R\left(T_{c}-T_{\infty }\right)}\right)exp\left(\frac{-K_{g}}{T_{c}\Delta Tf}\right)$$

where *K_g_* is the nucleation constant; Δ*T* is the supercooling difined by $T_{m}^{0}-T_{c}$, $T_{m}^{0}$ is the equilibrium melting point, here the value of $T_{m}^{0}=469.6\;K$ is used according to our previous paper [7]; *f* is a factor given as 2*T_c_/*($T_{m}^{0}+T_{c}$). U* = 1500 cal·mol^−1^ represents the activation energy for segment diffusion to the site of crystallization; *R* is the gas constant; $T_{\infty }$ = *T_g_* − 50 K is the Vogel–Fulcher–Tamman–Hesse (VFTH) parameters describing the transport of polymer segments across the liquid or crystal interface; and *G*_0_ is the front factor.

**Figure 8:**
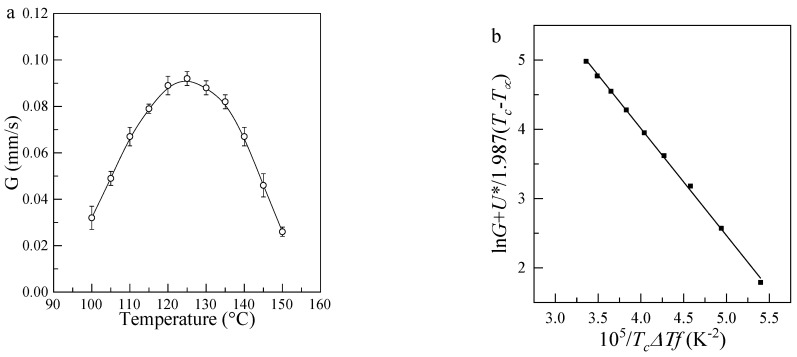
(**a**) Growth rate G (μm·s^−1^) as a function of *T_c_* for PLLA films; (**b**) Changes in *lnG* + U*/*R*(*T_c_* − *T*_∞_) as a function of 1/*T_c_*Δ*Tf*.

As shown in Figure 8b, by taking the logarithm of Equation (4), the *lnG* + U*/*R*(*T*_c_ − *T*_∞_) changes linearly with 1/*T_c_*Δ*Tf*, which we can obtain the values of *K_g_* and *lnG*_0_ from the slope and intercept, respectively. To obtain the sufficient direct information on the growth front of PLLA spherulites, we calculated the surface free energies σσ_e_ by further analyzing the nucleation constant *K_g_*.

The nucleation parameter *K_g_* is given as

(6) $$K_{g}=\frac{yb\sigma \sigma_{e}T_{m}^{0}}{\Delta h_{f}k_{B}}$$

where the variable y reflects the regime behavior and y = 4 for regimes I and III, and 2 for regime II. *b* is the layer thickness; *σ* is the lateral surface free energy; *σ_e_* is the fold surface free energy; Δ*h_f_* is the heat of fusion per unit volume; and *k_B_* Boltzmann’s constant. Here, the value of y=2 was used due to the explored *T_c_* ranging in the regime II, Δ*h_f_* = 174 × 10^6^ J·m^−3^ was used and the value of the layer thickness should be taken as 0.53 nm for PLLA crystals based on the previous report [37,38].

The value of σσ_e_ estimated is about 745 × 10^6^ J^2^·m^−4^, similar to the results reported by Vasanthakumari and Pennings [39]. We then estimated the fold surface free energy, *σ_e_* could to be 61.9 × 10^3^ J·m^−3^, by using the value of the lateral surface free energy, *σ* = 12.03 × 10^3^ J·m^−3^, obtained by Vasanthakumari and Pennings [39]. Although Vasanthakumari and Pennings calculated the *σ_e_* with low molecular weight, the similar value of *σ_e_* shows that fold surface at growth front for PLLA is independent on the molecular weight, which is verified by Pan et al. [40] who suggested that chains folding processing was dependent on the *T_c_*, regardless of the molecular weight.

## 4. Conclusions

Through the present work, the morphology, structure and isothermal crystallization kinetics of PLLA were explored at different crystallization temperatures through a combination of analytical techniques (POM and WAXD). It was found that, during the isothermal crystallization, the mechanism of crystallization of PLLA changes with temperature and mixed growth mechanism (two dimensional and three-dimensional growth of crystals) was present, resulting in the decrease of Avrami exponent *n*. At low or high temperatures, the crystallization rate is observed to be slower, apparently stemming from to the slow growth rate, or nucleation rate. Therefore, this work opens up potential avenues for the design of degradable PLLA products, with tunable physical attributes, for future medical and other environmentally friendly applications.

## Figures and Tables

**Table 1:** Atomic contents of elements.

Elements.	Atomic Number	Atomic Content [%]	Abs. Error [%] (1sigma)
C	6	68.02	7.21
O	8	31.67	4.93
Cr	24	0.27	0.12
Ni	28	0.04	0.11

**Table 2:** Isothermal crystallization parameters of PLLA.

*T_c_* (°C)	*n*	*t*_1/2_ (*s*)	*ln K*
100	2.8 ± 0.02	407	−17.1 ± 0.05
110	2.8 ± 0.01	540	−18.2 ± 0.03
120	2.5 ± 0.01	1133	−17.7 ± 0.07

## References

[1] Lim L.T., Auras R., Rubino M. (2008). Processing technologies for poly(lactic acid). Prog. Polym. Sci..

[2] Gazzotti S., Farina H., Lesma G., Rampazzo R., Piergiovanni L., Ortenzi M.A., Silvani A. (2017). Polylactide/cellulose nanocrystals: The in situ polymerization approach to improved nanocomposites. Eur. Polym. J..

[3] Androsch MLDLR (2018). Industrial Applications of Poly(lactic acid).

[4] Nobes G.A.R., Marchessault R.H., Chanzy H., Briese B.H., Jendrossek D. (1996). Splintering of Poly(3-hydroxybutyrate) Single Crystals by PHB-Depolymerase A from Pseudomonas lemoignei. Macromolecules.

[5] Hocking P.J., Marchessault R.H., Timmins M.R., Lenz R.W., Fuller R.C. (1996). Enzymatic Degradation of Single Crystals of Bacterial and Synthetic Poly(β-hydroxybutyrate). Macromolecules.

[6] Dai S., Jiang N., Ning Z., Gan Z. (2021). Relationship between crystallization state and degradation behavior of poly(l-lactide)/fourarmed poly(d,l-lactide)-block-poly(d-lactide) blends with different poly(d-lactide) block lengths. Polym. Int..

[7] Zhang R.-C., Sun D., Lu A., Zhong M., Xiong G., Wan Y. (2017). Equilibrium Melting Temperature of Polymorphic Poly(l-lactide) and Its Supercooling Dependence on Growth Kinetics. Polymers.

[8] Di Lorenzo M.L., Androsch R. (2019). Influence of α′-/α-crystal polymorphism on properties of poly(l-lactic acid). Polym. Int..

[9] Xu J.Z., Chen T., Yang C.L., Li Z.M., Mao Y.M., Zeng B.Q., Hsiao B.S. (2010). Isothermal Crystallization of Poly(l-lactide) Induced by Graphene Nanosheets and Carbon Nanotubes: A Comparative Study. Macromolecules.

[10] Mahendrasingam A., Blundell D.J., Parton M., Wright A.K., Rasburn J., Narayanan T., Fuller W. (2005). Time resolved study of oriented crystallisation of poly(lactic acid) during rapid tensile deformation. Polymer.

[11] Huang Y.-F., Kao H.-L., Ruan J., Su A.-C. (2010). Effects of Solution Status on Single-Crystal Growth Habit of Poly(l-lactide). Macromolecules.

[12] Ru J.-F., Yang S.-G., Zhou D., Yin H.-M., Lei J., Li Z.-M. (2016). Dominant β-Form of Poly(l-lactic acid) Obtained Directly from Melt under Shear and Pressure Fields. Macromolecules.

[13] Jalali A., Huneault M., Elkoun S. (2016). Effect of thermal history on nucleation and crystallization of poly(lactic acid). J. Mater. Sci..

[14] Di Lorenzo M.L., Rubino P., Immirzi B., Luijkx R., Hélou M., Androsch R. (2015). Influence of chain structure on crystal polymorphism of poly(lactic acid). Part 2. Effect of molecular mass on the crystal growth rate and semicrystalline morphology. Colloid Polym. Sci..

[15] Huang S., Li H., Jiang S. (2019). Crystal structure and unique lamellar thickening for poly(l-lactide) induced by high pressure. Polymer.

[16] Yuan C., Xu Y., Yang K., Wang Y., Wang Z., Cheng X., Su L. (2018). Isothermally crystallization behavior of poly (l-lactide) from melt under high pressure. Polym. Adv. Technol..

[17] Xie Q., Bao J., Shan G., Bao Y., Pan P. (2019). Fractional Crystallization Kinetics and Formation of Metastable β-Form Homocrystals in Poly(l-lactic acid)/Poly(d-lactic acid) Racemic Blends Induced by Precedingly Formed Stereocomplexes. Macromolecules.

[18] Kenji W., Jiro K. (2020). Extended-chain crystallization and stereocomplex formation of polylactides in a Langmuir monolayer. Polym. J..

[19] Chen D., Lei L., Zou M., Li X. (2021). Non-Isothermal Crystallization Kinetics of Poly(Ethylene Glycol)–Poly(l-lactide) Diblock Copolymer and Poly(Ethylene Glycol) Homopolymer via Fast-Scan Chip-Calorimeter. Polymers.

[20] Lv T., Li J., Huang S., Wen H., Li H., Chen J., Jiang S. (2021). Synergistic effects of chain dynamics and enantiomeric interaction on the crystallization in PDLA/PLLA mixtures. Polymer.

[21] Zhao L.-S., Cai Y.-H. (2020). Non-isothermal Crystallization, Melting Behavior, Thermal Decomposition, Fluidity and Mechanical Properties of Melt Processed Poly(l-lactic acid) Nucleated by *N*,*N*′-Adipic Bis(piperonylic acid) Dihydrazide. Polym. Sci. Ser. A.

[22] Banpean A., Sakurai S. (2021). Confined crystallization of Poly(ethylene glycol) in spherulites of Poly(l-lactic acid) in a PLLA/PEG blend. Polymer.

[23] Pan P., Yang J., Shan G., Bao Y., Weng Z., Cao A., Yazawa K., Inoue Y. (2012). Temperature-Variable FTIR and Solid-State 13C NMR Investigations on Crystalline Structure and Molecular Dynamics of Polymorphic Poly(l-lactide) and Poly(l-lactide)/Poly(d-lactide) Stereocomplex. Macromolecules.

[24] De Santis P., Kovacs A.J. (1968). Molecular conformation of poly(s-lactic acid). Biopolymers.

[25] Cartier L., Okihara T., Ikada Y., Tsuji H., Puiggali J., Lotz B. (2000). Epitaxial crystallization and crystalline polymorphism of polylactides. Polymer.

[26] Di Lorenzo M.L. (2005). Crystallization behavior of poly(l-lactic acid). Eur. Polym. J..

[27] Tsuji H., Tezuka Y., Saha S.K., Suzuki M., Itsuno S. (2005). Spherulite growth of l-lactide copolymers: Effects of tacticity and comonomers. Polymer.

[28] Chen Y.H., Zhong G.J., Lei J., Li Z.M., Hsiao B.S. (2011). In Situ Synchrotron X-ray Scattering Study on Isotactic Polypropylene Crystallization under the Coexistence of Shear Flow and Carbon Nanotubes. Macromolecules.

[29] Xie X.L., Sang Z.H., Xu J.Z., Zhong G.J., Li Z.M., Ji X., Wang R., Xu L. (2017). Layer structure by shear-induced crystallization and thermal mechanical properties of injection-molded poly(l-lactide) with nucleating agents. Polymer.

[30] Zaldua N., Liénard R., Josse T., Zubitur M., Mugica A., Iturrospe A., Arbe A., De Winter J., Coulembier O., Müller A.J. (2018). Influence of Chain Topology (Cyclic versus Linear) on the Nucleation and Isothermal Crystallization of Poly(l-lactide) and Poly(d-lactide). Macromolecules.

[31] Zhao L., Kong J. (2014). Isothermal crystallization of poly(l-lactide) and poly(butylene adipate) crystalline/crystalline blends. Polym. J..

[32] Avrami M. (1939). Kinetics of Phase Change. I General Theory. J. Chem. Phys..

[33] Avrami M. (1940). Kinetics of Phase Change. II Transformation-Time Relations for Random Distribution of Nuclei. J. Chem. Phys..

[34] Avrami M. (1941). Granulation, Phase Change, and Microstructure Kinetics of Phase Change III. J. Chem. Phys..

[35] Lorenzo A.T., Arnal M.L., Albuerne J., Müller A.J. (2007). DSC isothermal polymer crystallization kinetics measurements and the use of the Avrami equation to fit the data: Guidelines to avoid common problems. Polym. Test..

[36] Hoffman J.D., Davis G.T., Lauritzen J.I., Hannay N.B. (1976). Treatise on Solid State Chemistry.

[37] Miyata T., Masuko T. (1997). Morphology of poly(l-lactide) solution-grown crystals. Polymer.

[38] Miyata T., Masuko T. (1998). Crystallization behaviour of poly(l-lactide). Polymer.

[39] Vasanthakumari R., Pennings A.J. (1983). Crystallization kinetics of poly(l-lactic acid). Polymer.

[40] Pan P., Kai W., Zhu B., Dong T., Inoue Y. (2007). Polymorphous Crystallization and Multiple Melting Behavior of Poly(l-lactide): Molecular Weight Dependence. Macromolecules.

